# A Comparison of the First 60 Enucleation Cases Using a Thulium Fiber Laser without a Mentor to a Transurethral Resection of the Prostate (TURP) and Open Prostatectomy, and the Learning Curve

**DOI:** 10.3390/medicina60081356

**Published:** 2024-08-20

**Authors:** Ender Cem Bulut, Burak Elmas, Murat Yavuz Koparal, Çağrı Coşkun, Uğur Aydın, Kadir Şerefhan Erten, Serhat Çetin, Sabuhi Alishov, Ali Atan, Süleyman Yeşil, Bora Küpeli

**Affiliations:** 1 Urology Department, School of Medicine, Gazi University, Ankara 06560, Türkiye; elms.burak@gmail.com (B.E.); drmykoparal@gmail.com (M.Y.K.); serefhan96@gmail.com (K.Ş.E.); scetin86@yahoo.com (S.Ç.); aliatanpitt@hotmail.com (A.A.); syesil2003@yahoo.com (S.Y.); borakupeli@yahoo.com (B.K.); 2 Urology Department, Ağrı Training and Research Hospital, Ağrı 04200, Türkiye; drcagricoskun@gmail.com (Ç.C.); ugurr.aydinn@hotmail.com (U.A.); 3 Urology Department, Parkhayat Kütahya Hospital, Kütahya 43020, Türkiye; sabuhialishov@gmail.com

**Keywords:** benign prostatic hyperplasia, enucleation, prostatectomy, thulium fiber laser, ThuFLEP

## Abstract

*Background and Objectives*: In the surgical treatment of benign prostatic hyperplasia (BPH), laser enucleation of the prostate is recommended as an alternative to transurethral resection (TURP) and open prostatectomy (OP). The thulium fiber laser, with its superficial penetration depth, can offer a rapid learning process by causing less heat injury and capsule damage. This study compares the first 60 cases of an endourologist performing thulium fiber enucleation of the prostate (ThuFLEP) without a mentor to the results of OP and TURP performed by experienced surgeons. It also identifies the case number at which the operation time for ThuFLEP starts to plateau. *Materials and Methods*: Between 1 November 2021 and 1 November 2023, the initial 60 ThuFLEP cases of an endourologist with no prior enucleation experience were compared with TURP and OP operations performed by experienced surgeons. Since the first 60 ThuFLEP cases involved 80–120 cc prostates, TURP and OP operations within this size range performed during the same period were included in the study. The groups were assessed for age, preoperative and postoperative prostate volume, PSA levels, the IPSS, the IPSS Quality of Life (QoL), and maximum urinary flow (Q_max_). The 60 consecutive ThuFLEP cases were divided into three groups of 20 (Groups 1, 2, and 3) and compared for operation time, IPSS, and Q_max_. *Results*: The operation time for TURP was shorter than for ThuFLEP and OP (*p* < 0.001). There was no significant difference between ThuFLEP and OP in postoperative Q_max_ and IPSS, while TURP had lower values than the other two methods. For ThuFLEP, the operation time was longer in the first 20 cases but plateaued in groups 2 and 3 (*p* < 0.001). Postoperative Q_max_ and IPSS values showed no significant differences among the three ThuFLEP groups (*p* > 0.05). *Conclusions*: For large prostates, ThuFLEP provides better postoperative results than TURP and offers shorter catheterization and hospital stay times than OP. Its short learning curve makes it a preferable method for treating BPH compared to other laser techniques.

## 1. Introduction

Although open prostatectomy (OP) remains a preferred surgical approach for benign prostatic hyperplasia (BPH) patients with prostate volumes over 80 cc, minimally invasive procedures such as transurethral laser therapy—encompassing enucleation, vaporization, and resection—have been added alongside transurethral resection of the prostate (TURP). Enucleation is recommended as an alternative to both OP and TURP for all prostate sizes [1,2].

Despite being an alternative treatment for lower urinary tract symptoms caused by BPH for nearly 20 years, prostate enucleation is utilized by a limited number of centers, likely due to its prolonged learning curve. It is estimated that 50–60 cases are necessary to reach a plateau in the learning curve, depending on whether the training is through a mentor or video observation [3,4].

Different laser energy sources have been used for endoscopic enucleation in recent years. Following holmium-YAG and thulium-YAG lasers, thulium fiber lasers (TFLs) have been added to these energy sources. With a wavelength of 1940 nm, the TFL is absorbed by tissue at a much higher rate than other energy sources. This results in TFLs having minimal tissue penetration depth, providing more precise cuts with lower tissue damage [5]. Indeed, studies have shown that thulium fiber enucleation of the prostate (ThuFLEP) is similar in efficacy and efficiency to holmium laser enucleation of the prostate (HoLEP) [6,7,8]. Furthermore, enucleation with a TFL is argued to have a shorter learning curve than HoLEP [3,9,10].

This study aims to evaluate the effectiveness and outcomes of the first 60 consecutive enucleation cases performed by an endourologist using a TFL without a mentor, comparing the results with those of TURP and OP. Additionally, the number of cases required to reach the learning curve plateau has been investigated.

## 2. Materials and Methods

Between 1 November 2021 and 1 November 2023, the data of OP and TURP cases performed in our clinic, as well as the first 60 consecutive prostate enucleation cases performed by an endourologist (ECB), were retrospectively analyzed. A thulium fiber laser (TFL) was used in all prostate enucleation cases, which were the surgeon’s first enucleation operations. Since the first 60 cases involved prostates of 80–120 cc, TURP and OP operations within this size range performed during the same period were included in the study. The surgeon did not have an experienced mentor for enucleation and learned the operative techniques through videos. A total of 60 patients who underwent ThuFLEP, 88 patients who underwent OP, and 81 patients who underwent TURP were included in the study.

The inclusion criteria were having lower urinary tract symptoms due to BPH, not responding to medical treatment or experiencing increased symptom severity during treatment, urinary retention, recurrent hematuria, recurrent urinary tract infections, or renal impairment with bilateral hydronephrosis. Additionally, patients needed to have a prostate size of 80–120 cc to be included in the study.

Exclusion criteria were defined as having a history of previous prostate/urethral surgery, prostate cancer, a history of pelvic radiotherapy, or undergoing simultaneous lower urinary tract surgery (such as internal urethrotomy, lithotripsy, or transurethral resection of bladder tumors) alongside BPH surgery.

Patient data including age, preoperative and postoperative 3-month prostate volume, prostate-specific antigen (PSA) levels, the International Prostate Symptom Score (IPSS), the IPSS Quality of Life (QoL), maximum urine flow rate (Q_max_), postvoid residual urine (PVRU), perioperative operation time, the weight of the specimen, length of catheterization and hospital stay, hemoglobin drop (g/dL), and transfusion requirements were recorded. Complications occurring within 30 days post-operation were evaluated and classified according to the Clavien–Dindo system.

For TURP and OP, data from patients treated by experienced surgeons were used, while for ThuFLEP, data from an endourologist (ECB) who was new to the enucleation procedure and using TFLs were utilized. Patients who underwent ThuFLEP were designated as Group A, those who underwent OP as Group B, and those who underwent TURP as Group C. The first 20 consecutive ThuFLEP patients were designated as Group 1, the second 20 as Group 2, and the last 20 as Group 3.

### 2.1. Surgical Technique

Prostate enucleation was performed using a 26 F resectoscope (Olympus OES Pro laser resectoscopes, Olympus EMEA, Hamburg, Germany) that has a separate channel for the fiber and provides continuous flow. A TFL with a 550 nm fiber was used for the enucleation. An energy setting of 60 watts was used for cutting and 20 watts for coagulation. The enucleation was conducted using en-bloc enucleation with the early apical release technique, a method that has been previously established [11].

A HAWK SHAVER YSB-III morcellator (Hangzhou HAWK Optical Electronic Instruments Co., Ltd., Hangzhou, China) was used for morcellation. After morcellation, bleeding vessels were coagulated with the laser, and the procedure was concluded. A three-way 20 or 22 F urethral catheter was placed postoperatively. The surgical duration for ThuFLEP was calculated from the insertion of the resectoscope into the urethra until the urethral catheter was placed and the patient was handed over from anesthesia. Normal saline was used for irrigation during the operation. Continuous bladder irrigation was applied until the morning of the first postoperative day. The decision to remove the catheter was based on the absence of gross hematuria or clot retention. The attending physician decided on the patient’s discharge based on urine color.

For bipolar TURP, an OLYMPUS ESG-400 electrosurgical generator (Olympus Medical Systems, Center Valley, PA, USA) with settings of 200 watts for cutting and 120 watts for coagulation was used. The prostate was resected from the verumontanum to the bladder neck using a loop. Only the loop and ball electrodes were used during the operation. Saline was used for continuous irrigation. Tissue retrieval was performed using a Karl Storz REINER-ALEXANDER Syringe (KARL STORZ, Tuttlingen, Germany), a 150 mL syringe. A 20–22 F three-way Foley catheter was placed postoperatively. Continuous bladder irrigation was applied until the morning of the first postoperative day. The decision to remove the catheter was based on the absence of gross hematuria or clot retention. The attending physician decided on the patient’s discharge based on urine color.

For all three operations, patient discharge did not await catheter removal.

After reaching the Retzius space through a sub-umbilical median or Pfannenstiel incision in the OP operation, the bladder was opened at the anterior midline after placing two suspension sutures. After marking the bladder neck 360° with a scalpel, the prostate adenoma was enucleated with a finger. Control sutures were placed on the bladder neck at 3 and 9 o’clock, and then a 20–22 F three-way Foley catheter was inserted. The bladder was closed in two layers. A drain was placed in the lodge, and the wound was closed according to the anatomical layers. The decision to remove the catheter was based on the absence of gross hematuria or clot retention. The decision for patient discharge was made by the attending physician based on the color of the urine.

The efficacy and safety outcomes of the three groups of patients who underwent ThuFLEP, TURP, and OP were statistically compared.

This study was reviewed and approved by the Ethics Committee of Gazi University on 16 April 2024 (approval number:655).

### 2.2. Statistical Analysis

Statistical analysis was carried out using SPSS software (the Statistical Package for the Social Science, version 23, Armonk, New York, NY, USA). Statistical significance was set to *p* < 0.05. The normality of continuous data was checked using the Kolmogorov–Smirnov test. Continuous variables were reported as the median (min–max) and categorical variables as a number (percentage). The Kruskal–Wallis test was used for the statistical analysis of continuous variables. Dunn’s test with Bonferroni correction was applied for post hoc analyses. The chi-squared test was used for categorical data.

## 3. Results

There was no statistically significant difference between Groups A, B, and C in terms of age, preoperative IPSS scores, IPSS QoL scores, Q_max_ values, and PVRU values (*p* = 0.368, *p* = 0.606, *p* = 0.605, *p* = 0.685, and *p* = 0.575, respectively) (Table 1).

The median preoperative prostate volumes for Groups A, B, and C were 90 (80–120), 108 (87–120), and 84 (80–107), respectively, while the median preoperative PSA values were 4.2 (3.7–5.6), 5.3 (3.4–5.9), and 3.5 (3.4–4.5), respectively. There was a statistically significant difference between the groups in terms of preoperative prostate volumes and preoperative PSA values (*p* < 0.001 and *p* < 0.001, respectively). All groups were significantly different from each other for both parameters. (For preoperative prostate volumes and PSA values between Group A and Group B, Group A and Group C, Group B and Group C, values were *p* < 0.001, *p* = 0.001, *p* < 0.001, *p* < 0.001, *p* < 0.001, and *p* < 0.001, respectively) (Table 1).

The median operation times for Groups A, B, and C were 130 (40–180), 110 (60–160), and 100 (50–130) minutes, respectively; the median weights of tissue specimens were 73 (65–98), 89 (72–99), and 53 (50–67) grams; the median catheterization times were 1 (1–5), 5 (5–9), and 4 (3–8) days; and the median Hb level decrease was 1.3 (0.1–2.8), 2.9 (1.6–4.4), and 2.1 (0.8–3.6) g/dL, respectively. There was a statistically significant difference between the groups regarding operation time, weight of tissue specimens, catheterization time, and Hb level decrease (*p* < 0.001, *p* < 0.001, *p* < 0.001, and *p* < 0.001, respectively). All groups were significantly different from each other for these four parameters. (For operation time, weight of tissue specimen, catheterization time, and Hb level decrease, between Group A and Group B, Group A and Group C, Group B and Group C, values were *p* = 0.042, *p* < 0.001, *p* = 0.015, *p* < 0.001, *p* < 0.001, *p* < 0.001, *p* < 0.001, *p* < 0.001, *p* < 0.001, *p* < 0.001, *p* < 0.001, and *p* < 0.001, respectively) (Table 2).

The median resection speeds for Groups A, B, and C were 0.6 (0.4–1.8), 0.8 (0.5–1.6), and 0.6 (0.4–1.1) g/min, respectively; the median hospital stays were 1 (1–5), 5 (4–9), and 1 (1–6) days, respectively. There was a statistically significant difference between the groups regarding resection speed and hospital stay (*p* < 0.001 and *p* < 0.001, respectively). For both parameters, the difference was found to be due to the differences between Group A and Group B, and Group B and Group C. (For resection speed and hospital stay, between Group A and Group B, and Group B and Group C, values were *p* < 0.001, *p* < 0.001, *p* < 0.001, and *p* < 0.001, respectively) (Table 2).

There was no statistically significant difference between Groups A, B, and C in terms of transfusion rates, Clavien–Dindo grade I and grade II early complications, SUI, and urethral stricture or bladder neck contraction (*p* = 0.564, *p* = 0.775, *p* = 0.159, *p* = 0.354, and *p* = 0.126, respectively) (Table 2).

The median postoperative prostate volumes for Groups A, B, and C were 11 (7–24), 13 (7–24), and 18 (9–28) mL, respectively; the median postoperative PSA values were 0.7 (0.5–1.3), 2 (0.7–1.5), and 1.1 (0.5–1.6) ng/mL; and the median PSA decrease rates were 85.2% (70.9–89.3), 81.5% (65.8–86.7), and 69.8% (55.6–84.5), respectively. There was a statistically significant difference between the groups regarding postoperative prostate volumes, postoperative PSA values, and PSA decrease rates (*p* < 0.001, *p* < 0.001, and *p* < 0.001, respectively). All groups were significantly different from each other for these three parameters. (For postoperative prostate volumes, PSA values, and PSA decrease rates between Group A and Group B, Group A and Group C, Group B and Group C, values were *p* = 0.001, *p* < 0.001, *p* < 0.001, *p* < 0.001, *p* < 0.001, *p* = 0.011, *p* = 0.001, *p* < 0.001, and *p* < 0.001, respectively) (Table 3).

The median postoperative IPSS values for Groups A, B, and C were 5.5 (3–14), 6 (3–14), and 7 (4–15), respectively; the median postoperative Q_max_ values were 18 (13–29.1), 18.2 (13.2–30.2), and 14.4 (10.4–26.0) mL/s, respectively; and the median postoperative PVRU values were 10 (0–40), 20 (0–30), and 10 (0–40) mL, respectively. There was a statistically significant difference between the groups regarding postoperative IPSS, Q_max_, and PVRU (*p* < 0.001, *p* < 0.001, and *p* = 0.001, respectively). For these three parameters, the difference was found to be due to differences between Group A and Group C, and Group B and Group C (For postoperative IPSS, Q_max_, and PVRU, between Group A and Group C, and Group B and Group C, values were *p* < 0.001, *p* = 0.018, *p* < 0.001, *p* < 0.001, *p* = 0.02, and *p* = 0.04, respectively) (Table 3).

The median operation times for Groups 1, 2, and 3 were 140 (120–180), 115 (70–140), and 115 (40–150) minutes, respectively.

There was a statistically significant difference between the groups regarding operation time (*p* < 0.001). The difference for this parameter was found to be due to differences between Group 1 and Group 2, and Group 1 and Group 3 (*p* < 0.001 and *p* = 0.001, respectively) (Table 4).

There was no statistically significant difference between Groups 1, 2, and 3 in terms of postoperative IPSS, postoperative Q_max_, and postoperative IPSS QoL (*p* = 0.220, *p* = 0.978, and *p* = 0.935, respectively) (Table 4).

## 4. Discussion

First described in 1983, enucleation has become a standard method in treating patients with BPH due to the identification of laser and bipolar energy, the detection of lower complication rates compared to the other two methods, and its applicability to all sizes [10,12,13,14].

Previous studies have shown that patients who underwent prostate enucleation had similar functional outcomes to those who underwent TURP and OP. Still, enucleation provided lower complication rates and shorter hospital stays [15,16,17,18]. Herden et al., in their study with a high number of patients comparing enucleation with TURP and OP, reported that the hospital stay was shorter in enucleation compared to the other two methods. At the same time, the transfusion rate was higher in OP [19]. In our study, while the hospital stay was higher in OP, the reason for the similarity between TURP and ThuFLEP might be that the removal of the urethral catheter is not awaited for the discharge of TURP patients. Indeed, despite the differences in catheterization times, the hospital stays are similar.

Although open prostatectomy has high efficacy for large prostates, it is associated with high postoperative complications and morbidity rates. Therefore, despite its steep learning curve, enucleation is becoming increasingly preferred [20,21]. In the study by Enikeev et al. comparing the results of OP and ThuFLEP, it was argued that while both methods are highly effective for large prostates (>80 cc), ThuFLEP is a more preferable method due to its lower complication and transfusion rates. They also suggested that thulium fiber might be advantageous over other laser sources due to its reliable hemostasis and shallower penetration depth [22]. In our study, IPSS scores were similar between OP and ThuFLEP. Similar outcomes can be expected since the transitional zone is theoretically wholly removed in both methods. It should also be considered that while the surgeons performing OPs were experienced, ThuFLEP was performed by a surgeon inexperienced in enucleation.

In another study by Enikeev et al., they compared the efficacy and complication rates of TURP and ThuFLEP in prostates <80 cc. In this study, they reported that ThuFLEP had shorter operation, catheterization, and hospital stay times compared to TURP, and that the reduction in prostate volume and PSA levels, as well as the long-term increase in Q_max_, were higher [23]. Unlike their study, our study evaluated larger prostates. However, it can be concluded that ThuFLEP, even when performed by a surgeon in the learning phase, is a more effective method for large prostates in terms of postoperative prostate volume, PSA decrease, and functional outcomes (IPSS, Q_max_) compared to TURP.

The trifecta of BPH enucleation includes defining the enucleation plane with good anatomical dissection, achieving sufficient hemostasis to prevent bleeding-related complications, and avoiding urethral/capsular injuries [24]. However, the significant barriers to the widespread adoption of HoLEP include its high cost and steep learning curve [18,25]. Brunckhorst et al. reported in their study on the HoLEP learning curve that the enucleation rate plateaued after 50–60 cases [3]. The TFL has advantages over Ho-YAG and Tm-YAG in enucleation. These advantages include precise and rapid incisions due to the wave mode, less heat injury and potential capsule damage due to a shallower penetration depth, and preservation of the enucleation plane due to low carbonization [7,10,26,27,28]. With these features, TFLs can be seen as an ideal energy source at the beginning of prostate enucleation, providing advantageous features for the trifecta. Indeed, it is thought that the learning curve for enucleation with TFLs can be reduced to eight to sixteen cases, and the reduction in complication rates can prevent surgeons from abandoning the procedure [10,29].

**Figure 1:**
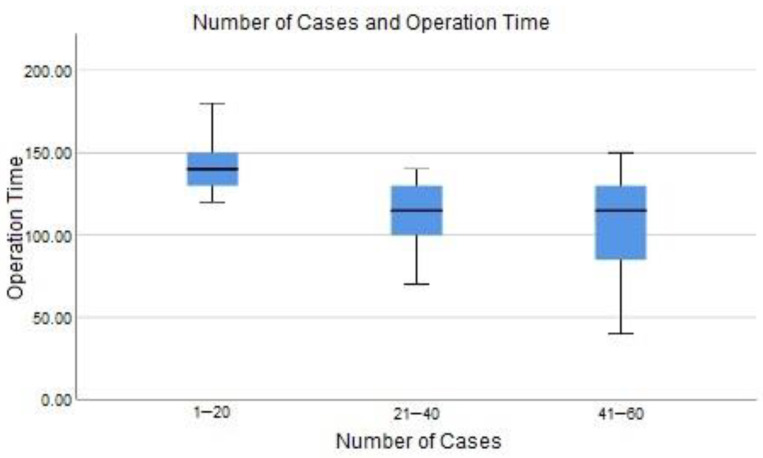
Learning curve graphs for operation time.

In our study, the plateau in operation time after the first 20 cases supports this observation. However, the lack of differences in functional outcomes in our results suggests that removing the adenoma is sufficient to achieve appropriate functional outcomes, even if it takes longer. In a multicenter and prospective study by Robert et al., the first 20 cases of surgeons new to enucleation were evaluated. They reported that at this number, surgeons were still unable to perform effective enucleation and that many surgeons tended to abandon the method due to the operation time and procedural difficulty [4]. The primary reason is the difficulty in defining the enucleation plane, the border between the adenoma and the peripheral zone, staying within this plane during the operation, and managing complications [10,29]. In our study, the operation times fell to acceptable levels after 20 cases, and enucleation became the primary method for treating BPH in the clinic without being abandoned, suggesting that TFL is more advantageous than other lasers during the learning phase (Figure 1). In a study evaluating the learning curves of ThuFLEP, HoLEP, and MEP (monopolar electroenucleation of the prostate), it was reported that the enucleation rate was slightly higher with ThuFLEP compared to the other two methods. However, these studies indicate that the learning curve can be shortened with mentor-assisted enucleation. Shin et al. compared the first 20 HoLEP cases performed by two groups of surgeons, one learning through self-study using videos and written information and the other learning with a mentor-assisted hand-grab technique, and reported that the group performing their first cases with a mentor had a better enucleation efficiency [30]. In our study, all enucleations were performed without a mentor, yet comparable results were obtained with TURP and OP performed by experienced surgeons. It was even concluded that there might be advantages in terms of hospital stay, catheterization time, and some postoperative outcomes.

While our study adds a new comparison to the limited number of studies in the literature comparing ThuFLEP, OP, and TURP, to our knowledge, it is the first to compare TFL enucleation without a mentor to these two methods and evaluate the learning curve. However, the study has significant limitations. The study is limited by its retrospective nature. The retrospective design led to the study being designed with limited data. Additionally, the short follow-up period of three months for enucleation patients prevented the evaluation of long-term functional outcomes, complications, and the need for reintervention. It is more appropriate to start enucleation during the learning period with prostates of moderate size, as it is known that optimal outcomes and complication rates may not be achieved with prostates smaller than 50–60 cc or larger than 100 cc. However, the prostates larger than 100 cc in our study may have influenced our ThuFLEP results. Since the cases only included 80–120 cc prostates, which are more suitable for inexperienced surgeons, the results can only be generalized to some sizes. Moreover, comparing the initial ThuFLEP results with HoLEP, in addition to TURP and OP, could have allowed for an assessment of the technical differences the thulium fiber laser provided. However, the lack of HoLEP in our clinic prevented this evaluation. The fact that the surgeons performing TURP and OP were experienced led to comparing the first 60 ThuFLEP cases with the results of TURP and OP performed by experienced surgeons. Furthermore, a cost-effectiveness analysis of these three methods could influence their preference.

## 5. Conclusions

In 80–120cc prostates, where all three methods can be preferred, ThuFLEP offers better postoperative outcomes than TURP and stands out for a shorter catheterization time than OP. Additionally, with its short learning curve, it could be preferable for BPH treatment compared to other laser methods. However, prospective studies with a larger patient population and the inclusion of the widely used HoLEP could provide more significant insights.

## Figures and Tables

**Table 1:** Patient characteristics and preoperative data.

	Group A (*n* = 60) Median (Min–Max)	Group B (*n* = 88) Median (Min–Max)	Group C (*n* = 81) Median (Min–Max)	*p*
Age, years (mean ± STD)	67.1 ± 7.1	68.9 ± 6.8	67.6 ± 7.1	0.368
Prostatic volume, cc	90 (80–120)	108 (87–120)	84 (80–107)	<0.001 ^*αβγ^
PSA preoperative, ng/mL	4.2 (3.7–5.6)	5.3 (3.4–5.9)	3.5 (3.4–4.5)	<0.001 ^*αβγ^
IPSS, score	23 (20–30)	23 (20–30)	22 (17–30)	0.606
IPSS QoL, score	4 (3–6)	5 (3–6)	4 (3–6)	0.605
Q_max_, mL/s	7.8 (4.1–13.7)	7.6 (3.1–12.6)	7.6 (3.8–13.9)	0.685
PVRU, mL	92.5 (30–230)	90 (10–230)	90 (10–220)	0.575

* Statistically significant difference. α: There is a statistically significant difference between ThuFLEP and OP (*p* < 0.05). β: There is a statistically significant difference between ThuFLEP and TURP (*p* < 0.05). γ: There is a statistically significant difference between OP and TURP (*p* < 0.05). ThuFLEP = thulium fiber laser enucleation of the prostate; OP = open prostatectomy; TURP = transurethral resection of prostate; PSA = prostate-specific antigen; IPSS = International Prostate Symptom Score; QoL = quality of life; Q_max_ = maximum urine flow rate; PVRU = postvoid residual urine. Group A: ThuFLEP, Group B: OP, Group C: TURP.

**Table 2:** Perioperative parameters and complications.

	Group A (*n* = 60) Median (Min–Max)	Group B (*n* = 88) Median (Min–Max)	Group C (*n* = 81) Median (Min–Max)	*p*
Operation time, min	130 (40–180)	110 (60–160)	100 (50–130)	<0.001 ^*αβγ^
Weight of tissue specimen, g	73 (65–98)	89 (72–99)	53 (50–67)	<0.001 ^*αβγ^
Resection speed, g/min	0.6 (0.4–1.8)	0.8 (0.5–1.6)	0.6 (0.4–1.1)	<0.001 ^*αγ^
Catheterization time, days	1 (1–5)	5 (5–9)	4 (3–8)	<0.001 ^*αβγ^
Hospital stay, days	1 (1–5)	5 (4–9)	1 (1–6)	<0.001 ^*αγ^
Hemoglobin level decrease, g/dL	1.3 (0.1–2.8)	2.9 (1.6–4.4)	2.1 (0.8–3.6)	<0.001 ^*αβγ^
Transfusion, *n* (%)	1 (1.7%)	4 (4.5%)	2 (2.5%)	0.564
Early Complication				
Clavien–Dindo I				0.775
Short-term SUI	7 (11.7%)	4 (4.5%)	7 (8.6%)	
Short-term UUI	3 (5%)	9 (10.2%)	5 (6.2%)	
Injury of bladder-wall	1 (1.7%)	0 (0%)	0 (0%)	
Injury of ureteral orifice	1 (1.7%)	1 (1.1%)	1 (1.2%)	
Clavien–Dindo II				0.159
Urinary tract infection	2 (3.3%)	8 (9.1%)	4 (4.9%)	
Acute urinary retention	1 (1.7%)	2 (2.3%)	4 (4.9%)	
Clot retention	1 (1.7%)	4 (4.5%)	8 (9.9%)	
Late Complication				
SUI *n* (%)	1 (1.7%)	6 (6.8%)	4 (4.9%)	0.354
Urethral stricture or bladder neck contraction *n* (%)	1 (1.7%)	1 (1.1%)	5 (6.2%)	0.126

* Statistically significant difference. α: There is a statistically significant difference between ThuFLEP and OP (*p* < 0.05). β: There is a statistically significant difference between ThuFLEP and TURP (*p* < 0.05). γ: There is a statistically significant difference between OP and TURP (*p* < 0.05). ThuFLEP = thulium fiber laser enucleation of the prostate; OP = open prostatectomy; TURP: transurethral resection of prostate; SUI: stress urinary incontinence; UUI: urge urinary incontinence. Group A: ThuFLEP, Group B: OP, Group C: TURP.

**Table 3:** Postoperative outcomes after 3 months.

	Group A (*n* = 60) Median (Min–Max)	Group B (*n* = 88) Median (Min–Max)	Group C (*n* = 81) Median (Min–Max)	*p*
Prostatic volume, cc	11 (7–24)	13 (7–24)	18 (9–28)	<0.001 ^*αβγ^
PSA postoperative, ng/mL	0.7 (0.5–1.3)	2 (0.7–1.5)	1.1 (0.5–1.6)	<0.001 ^*αβγ^
PSA decrease, %	85.2 (70.9–89.3)	81.5 (65.8–86.7)	69.8 (55.6–84.5)	<0.001 ^*αβγ^
IPSS, score	5.5 (3–14)	6 (3–14)	7 (4–15)	<0.001 ^*βγ^
IPSS QoL, score	1 (1–2)	1.5 (1–2)	2 (1–3)	0.087
Q_max_, mL/s	18 (13–29.1)	18.2 (13.2–30.2)	14.4 (10.4–26.0)	<0.001 ^*βγ^
PVRU, mL	10 (0–40)	20 (0–30)	10 (0–40)	0.001 ^*βγ^

* Statistically significant difference. α: There is a statistically significant difference between ThuFLEP and OP (*p* < 0.05). β: There is a statistically significant difference between ThuFLEP and TURP (*p* < 0.05). γ: There is a statistically significant difference between OP and TURP (*p* < 0.05). ThuFLEP = thulium fiber laser enucleation of the prostate; OP = open prostatectomy; TURP = transurethral resection of prostate; PSA = prostate-specific antigen; IPSS = International Prostate Symptom Score; QoL = quality of life; Q_max_ = maximum urine flow rate; PVRU = postvoid residual urine. Group A: ThuFLEP, Group B: OP, Group C: TURP.

**Table 4:** Comparison of operation time, postoperative Q_max_, IPSS, and IPSS QoL for consecutive cases.

	Group 1 (1–20) Median (Min-Max)	Group 2 (21–40) Median (Min-MAX)	Group 3 (41–60) Median (Min-Max)	*p*
Operation time, min	140 (120–180)	115 (70–140)	115 (40–150)	<0.001 ^*αγ^
Q_max_ postoperative, mL/s	19.8 (13–29.1)	17.7 (14.2–23.7)	17.9 (14.6–22.4)	0.220
IPSS postoperative, score	6 (3–10)	5 (3–10)	5.5 (3–14)	0.978
IPSS QoL postoperative, score	1 (1–2)	1 (1–2)	1 (1–2)	0.935

* Statistically significant difference. α: There is a statistically significant difference between Group 1 and Group 2 (*p* < 0.05). γ: There is a statistically significant difference between Group 2 and Group 3 (*p* < 0.05). IPSS = International Prostate Symptom Score; QoL = quality of life; Q_max_ = maximum urine flow rate.

## Data Availability

The data that support the findings of this study are available from the corresponding author upon reasonable request.
